# A convolutional neural network with self-attention for fully automated metabolic tumor volume delineation of head and neck cancer in $[^{18}$F]FDG PET/CT

**DOI:** 10.1007/s00259-023-06197-1

**Published:** 2023-04-20

**Authors:** Pavel Nikulin, Sebastian Zschaeck, Jens Maus, Paulina Cegla, Elia Lombardo, Christian Furth, Joanna Kaźmierska, Julian M. M. Rogasch, Adrien Holzgreve, Nathalie L. Albert, Konstantinos Ferentinos, Iosif Strouthos, Marina Hajiyianni, Sebastian N. Marschner, Claus Belka, Guillaume Landry, Witold Cholewinski, Jörg Kotzerke, Frank Hofheinz, Jörg van den Hoff

**Affiliations:** 1 Helmholtz-Zentrum Dresden-Rossendorf, PET Center, Institute of Radiopharmaceutical Cancer Research, Bautzner Landstrasse 400, 01328 Dresden, Germany; 2 Department of Radiation Oncology, Charité – Universitätsmedizin Berlin, corporate member of Freie Universität Berlin and Humboldt-Universität zu Berlin, Berlin, Germany; 3 Berlin Institute of Health at Charité – Universitätsmedizin Berlin, Berlin, Germany; 4 Department of Nuclear Medicine, Greater Poland Cancer Centre, Poznan, Poland; 5 Department of Radiation Oncology, University Hospital, Ludwig-Maximilians-University (LMU) Munich, Munich, Germany; 6 German Cancer Consortium (DKTK), Partner Site Munich, Munich, Germany; 7 Department of Nuclear Medicine, Charité – Universitätsmedizin Berlin, corporate member of Freie Universität Berlin and Humboldt-Universität zu Berlin, Berlin, Germany; 8 Department of Nuclear Medicine, University Hospital, Ludwig-Maximilians-University (LMU) Munich, Munich, Germany; 9 Department of Radiation Oncology, German Oncology Center, European University Cyprus, Limassol, Cyprus; 10 Electroradiology Department, University of Medical Sciences, Poznan, Poland; 11 Radiotherapy Department II, Greater Poland Cancer Centre, Poznan, Poland; 12 Department of Nuclear Medicine, University Hospital Carl Gustav Carus, Technische Universität Dresden, Dresden, Germany

**Keywords:** FDG PET, Metabolic tumor volume, MTV, Head and neck cancer, HNC, Convolutional neural network

## Abstract

**Purpose:**

PET-derived metabolic tumor volume (MTV) and total lesion glycolysis of the primary tumor are known to be prognostic of clinical outcome in head and neck cancer (HNC). Including evaluation of lymph node metastases can further increase the prognostic value of PET but accurate manual delineation and classification of all lesions is time-consuming and prone to interobserver variability. Our goal, therefore, was development and evaluation of an automated tool for MTV delineation/classification of primary tumor and lymph node metastases in PET/CT investigations of HNC patients.

**Methods:**

Automated lesion delineation was performed with a residual 3D U-Net convolutional neural network (CNN) incorporating a multi-head self-attention block. 698 $[^{18}$F]FDG PET/CT scans from 3 different sites and 5 public databases were used for network training and testing. An external dataset of 181 $[^{18}$F]FDG PET/CT scans from 2 additional sites was employed to assess the generalizability of the network. In these data, primary tumor and lymph node (LN) metastases were interactively delineated and labeled by two experienced physicians. Performance of the trained network models was assessed by 5-fold cross-validation in the main dataset and by pooling results from the 5 developed models in the external dataset. The Dice similarity coefficient (DSC) for individual delineation tasks and the primary tumor/metastasis classification accuracy were used as evaluation metrics. Additionally, a survival analysis using univariate Cox regression was performed comparing achieved group separation for manual and automated delineation, respectively.

**Results:**

In the cross-validation experiment, delineation of all malignant lesions with the trained U-Net models achieves DSC of 0.885, 0.805, and 0.870 for primary tumor, LN metastases, and the union of both, respectively. In external testing, the DSC reaches 0.850, 0.724, and 0.823 for primary tumor, LN metastases, and the union of both, respectively. The voxel classification accuracy was 98.0% and 97.9% in cross-validation and external data, respectively. Univariate Cox analysis in the cross-validation and the external testing reveals that manually and automatically derived total MTVs are both highly prognostic with respect to overall survival, yielding essentially identical hazard ratios (HR) ($\text {HR}_{\text {man}}=1.9$; $p<0.001$ vs. $\text {HR}_{\text {cnn}}=1.8$; $p<0.001$ in cross-validation and $\text {HR}_{\text {man}}=1.8$; $p=0.011$ vs. $\text {HR}_{\text {cnn}}=1.9$; $p=0.004$ in external testing).

**Conclusion:**

To the best of our knowledge, this work presents the first CNN model for successful MTV delineation and lesion classification in HNC. In the vast majority of patients, the network performs satisfactory delineation and classification of primary tumor and lymph node metastases and only rarely requires more than minimal manual correction. It is thus able to massively facilitate study data evaluation in large patient groups and also does have clear potential for supervised clinical application.

**Supplementary Information:**

The online version contains supplementary material available at 10.1007/s00259-023-06197-1.

## Introduction

Primary treatment approaches for localized head and neck cancer (HNC) include either definitive radiochemotherapy or surgery. The latter is often followed by adjuvant radiotherapy or radiochemotherapy. Treatment related side effects are considerable and differ between both primary treatment approaches, as shown by the randomized ORATOR trial in oropharyngeal carcinomas [1]. For primary radiochemotherapy, radiosensitivity differs considerably between individual patients and local tumor recurrences remain an important clinical issue. Biomarkers for an improved personalized treatment include quantitative PET parameters, notably metabolic tumor volume (MTV), total lesion glycolysis, and $\textrm{SUV}_\mathrm{{max}}$ of the primary tumor which have been shown to be prognostic of clinical outcome in patients with HNC [2–6]. Evaluation of lymph node (LN) metastases in addition to the primary tumor has potential to further increase the prognostic value of PET [7]. Such analysis requires, however, accurate delineation and classification of all lesions which is very time-consuming when performed manually. Additionally, the tumor volumes can be prone to interobserver variability which hampers reproducibility of the results. The problem of accelerating tumor delineation in PET has previously been addressed by several groups using semi-automated methods such as fixed or adaptive thresholding, fuzzy locally adaptive Bayesian segmentation, region growing method, etc. [8]. Such approaches provide satisfactory results at sufficiently high target to background contrast but become increasingly more inaccurate with decreasing contrast which makes manual intervention regularly necessary. Consequently, notable time demands are imposed on the user, especially for LN delineation.

The recent emergence of deep learning-based methods for medical image analysis [9–13] allowed for significant progress in the tasks of therapy response [14–19] and clinical outcome [20–28] prediction, image registration [29–34], exam and object classification [35–46], object detection [47–54], and, finally, object delineation [55–68]. More specifically, the approaches to HNC cancer lesion delineation mostly rely on similar U-Net-like architectures but differ regarding the choice of target volume definition, considered patient population, and employed imaging modalities. Some researchers have exclusively considered the morphological modalities, i.e. CT [69–74] and MRI [75–79] or a combination of both [80], with Dice similarity coefficients (DSCs) reaching 0.74 for primary tumor and 0.66 for LN metastases in CT and 0.65 for primary tumor and 0.58 for LN metastases in MRI. For the special case of MRI in nasopharyngeal cancer a much higher DSC of up to 0.90 has been reported [78]. Furthermore, many studies report that combining CT or MRI with PET improves the network’s performance considerably [70–74, 81] compared to usage of only a single modality. The majority of state-of-the art designs utilizes PET/CT which has been shown to be slightly superior to PET/MR [80] and also is much more widely available. Examples include primary tumor delineation in oropharyngeal cancer [72, 82, 83] (DSC = 0.61), primary tumor + LN metastases delineation in squamous cell carcinoma of the oral cavity, oropharynx, hypopharynx and larynx [73, 74, 80] (DSC = 0.75), and primary + LN metastases delineation in a non-specified HNC [70, 71, 81] (DSC = 0.82). The number of proposed solutions to the problem at hand increased drastically with creation of the HECKTOR challenge aiming on primary gross tumor volume (GTV) delineation in oropharyngeal cancer using a substantial PET/CT database [82]. The most recent challenge included contributions from 20 teams scoring DSCs of [0.63$-$0.78] [84]. Interestingly, despite a large variety of proposed solutions, the winning contribution relied on the well known 3D U-Net architecture with only minimal modifications [85].

It is important to emphasize that the above-mentioned studies aimed at GTV rather than MTV delineation and that these two volumes are not identical in general. MTV is mainly used in a diagnostic context and for therapy response assessment. Therefore, sensitivity and specificity should be well balanced to avoid overdiagnosis. In contrast, GTV is mainly utilized in radiotherapy planning where a higher sensitivity might be preferred at the expense of a lower specificity to reduce the risks of underdosage of malignant lesions. As a consequence, MTV might possess higher prognostic power compared to GTV [86]. So far, only a single study investigated the CNN-based fully automated MTV (PET-based GTV) delineation in HNC [87]. The authors considered multiple CNN architectures and loss functions in a population of 470 patients achieving DSC = 0.87, demonstrating generally comparable performance with different configurations. However, to the best of our knowledge the possibility of automated differentiation between primary tumors and LN metastases has not been thoroughly investigated so far. Therefore, our goal was development of an automated tool for MTV delineation and classification of primary tumor and lymph node metastases in HNC in PET/CT. Additionally, our aim was to compare the manually and CNN derived PET parameters regarding outcome prediction of patients in an independent external cohort of patients.

## Methods

### Patients and data acquisition

1133 patients available from a retrospective cohort of an ongoing clinical multicenter investigation [4] were considered for inclusion in the present study. Exclusion occurred as follows:

- no CT data sets: $N = 165$
- severe metal artifacts in the CT data sets: $N = 45$
- no sizable $[^{18}$F]FDG uptake, lesion identification/delineation in PET thus impossible: $N = 44$

Ultimately, 879 patients could thus be included in the current study.

The data were split into a main dataset used for training, validation, and testing and a dataset for external testing using only data from sites which were not included in network model generation. The main dataset consisted of 698 $[^{18}$F]FDG PET/CT scans of head and neck squamous cell carcinoma (HNSCC) patients (535 men and 163 women, mean age 61 years, range 25–87) from three clinical sites (Berlin, Germany ($N = 175$), Dresden, Germany ($N = 24$), Poznan, Poland ($N = 22$)) and 5 public databases (Data from Head-Neck-PET-CT [88] ($N = 269$), Data from Head-Neck-Radiomics-HN1 [89] ($N = 34$), Imaging and clinical data archive for head and neck squamous cell carcinoma patients treated with radiotherapy [90] ($N = 32$), Radiology Data from The Cancer Genome Atlas Head-Neck Squamous Cell Carcinoma [TCGA-HNSC] collection [91] ($N = 11$), Data From QIN-HEADNECK [92] ($N = 131$)). The dataset for external testing included $N = 15$ patients from Limassol, Cyprus and $N = 166$ patients from Munich, Germany (138 men and 43 women, mean age 63 years, range 28–89).

The main dataset included 643 primary tumors and 1078 LN metastases with mean (median) volumes of 13.21 (8.16) ml and 5.45 (2.74) ml, respectively. The external dataset contained 175 primary tumors and 397 LN metastases with mean (median) volumes of 15.62 (8.05) ml and 4.30 (0.70) ml, respectively. The most frequent localizations of primary tumor in the two datasets (main/external, respectively) were oropharynx (63%/41%), larynx (17%/17%), oral cavity (6%/19%), hypopharynx (6%/14%), and nasopharynx (6%/6%). The majority of the patients were staged UICC IV (69%/67%). Details on the respectively utilized PET/CT systems, data acquisition, and image reconstruction can be found in Supplementary Materials as well as in [4] and citations therein.

### Ground truth definition

The interactive lesion delineations performed in the context of the above-mentioned multicenter investigation served as ground truth for network training and evaluation. For this delineation, the metabolically active areas of, both, primary tumor and lymph node metastases were identified in the PET data by a semi-automatic algorithm based on adaptive thresholding considering the local background [93, 94] using the ROVER software (version 3.0.41; ABX GmbH, Radeberg, Germany). Each proposed region of interest (ROI) delineation was individually verified by one of two experienced observers and manually corrected (also using the ROVER software) where this was deemed necessary. For the primary tumor manual correction was required in 41 out of 879 patients. Manual correction was necessary more frequently in lymph nodes (716 out of 1475 lesions). The majority of corrections concerned lesions with diffuse low tracer accumulation. Furthermore, in a few patients where tumor and lymph nodes were in close vicinity, the ROVER algorithm was not able to generate separate ROIs and erroneously fused the neighboring lesions in a single ROI.

### Network architecture, data preprocessing, and training procedure

Automated lesion delineation was performed with a residual 3D U-Net CNN modified by inclusion of a Multi-Head Self-Attention (MHSA) block [95] at the bottom of the U-Net in order to improve global context awareness during lesion classification. More details on the CNN design are provided in Supplementary Materials. The proposed architecture was implemented using the Apache MXNet (version 1.9.0) package for the R language and environment for statistical computing (version 4.2.0) [96].

The network was trained using pairs of PET and CT volumes as input. The data were pre-processed as follows. First, all image volumes were resampled to a common voxel size of $2.5\times 2.5\times 2.5$ mm and centrally cropped to a matrix size of $128\times 128$ in the transaxial plane (corresponding to $32\times 32$ cm field coverage). A further variable axial crop was performed preserving the head and neck region of the respective PET/CT image volume. In the next step, image patches of size $128\times 128\times 32$ were extracted with a partial overlap of at least 75% in axial direction. After windowing the CT intensity values to a range of $[-150,150]$ HU, PET and CT volumes were individually normalized to the range [0, 1]. The ground truth delineations were encoded into one-hot format for the three classes — background, primary tumor, and LN — using a voxel grid matching the one holding the PET/CT data as described above. The whole process results in a total of 9535 data samples in the main dataset.

A 5-fold cross-validation scheme with 5 equally sized folds was employed in order to assess the network performance in all data in the main dataset. For each of the 5 training runs, 64% of the data were assigned for training, 16% for validation, and 20% for testing, respectively. Network training was performed for 200 epochs with the Adadelta optimizer (batch size = 16) using Dice + Cross-Entropy as loss function. The training process was monitored by calculating the soft DSC in the validation data. More details on the loss function and evaluation metric as well as the training logs are provided in Supplementary Materials. Training was stopped if no improvement in the evaluation metric was recorded for 30 epochs. The 5 models achieving the highest scores in the respective validation data were selected for further evaluation.

### Network evaluation

Each of the five resulting CNN models was used to predict primary tumor and LN metastases probability maps in its respective test subset of the main dataset. Since the network does not directly predict the probability for the whole image but only for image patches, the predictions for the entire image volume was derived from the predictions in the separate patches by calculating the output probability in the overlap areas as a weighted sum of probabilities. The weights were chosen to be 3D Gaussian with the respective full-widths at half-maximum equal to the patch side lengths. Such weighting is based on our empirical finding that the CNN predictions are more reliable in the center of the patch. Each voxel was assigned to a class (background: 0, primary tumor: 1, LN metastases: 2) according to the highest probability in the derived maps. Accordingly, the union of all voxels with class 1 (class 2) defines the primary tumor (LN metastases) ROI. ROIs with volumes $<0.1$ ml (both, manually and automatically delineated) were excluded from further analysis. Finally, the predictions obtained in the disjunct test data of the different folds were pooled, i.e., the complete available data set was considered for further analysis of network performance rather than analyzing in turn each of the folds separately. The complete data analysis for the present investigation was performed on the above-mentioned image volumes resampled to cubic (2.5 mm)$^3$ voxels that were processed by the CNN. It should be noted that for possible applications of the network beyond MTV determination (notably in the context of radiation treatment planning), it would be necessary to transform the CNN outputs back to the original voxel grid prior to the voxel class assignment.

The evaluation was additionally performed in an external test dataset to assess the capability of the network to generalize to data from so far unseen sources. Separate runs were performed with all 5 CNN models and class membership was determined using probability maps obtained by averaging of the individual model outputs.

### Spatial concordance

The spatial concordance between manual and automatic delineations was quantified using the DSC for primary tumor, LN metastases, and the union of all lesions representing the total tumor burden (TTB), respectively. We calculated, both, cohort DSC (determined for the union of all delineations across all patients) as well as individual DSC (determined for each patient) together with mean and median values of the corresponding distributions. Furthermore, the mean absolute difference between manual and automated TTB delineations as well as the corresponding correlation coefficient were computed. In these calculations, 1% of the data exhibiting the highest absolute TTB differences were rejected to reduce the influence of outliers.

### Classification capabilities

The network’s capability to distinguish between primary tumor and LN metastases was assessed by considering the subset of voxels included in both manual and CNN delineations. Voxels of primary tumor and LN metastases (as defined in manual delineation) which were correctly classified by the CNN were counted as true primary tumor ($\textrm{T}_\mathrm{{PT}}$) and true LN ($\textrm{T}_\mathrm{{LN}}$), respectively. Primary tumor voxels classified as LN metastases and LN metastases voxels classified as primary tumor were counted as false LN ($\textrm{F}_\mathrm{{LN}}$) and false primary ($\textrm{F}_\mathrm{{PT}}$), respectively. Classification performance was quantified by the true positive rate of “primary tumor” labeled voxels $\textrm{TPR}_\mathrm{{PT}} = \textrm{T}_\mathrm{{PT}}/(\textrm{T}_\mathrm{{PT}} + \textrm{F}_\mathrm{{LN}})$, the corresponding true positive rate $\textrm{TPR}_\mathrm{{LN}} = \textrm{T}_\mathrm{{LN}}/(\textrm{T}_\mathrm{{LN}} + \textrm{F}_\mathrm{{PT}})$ of “lymph node metastasis” labeled voxels, and the classification accuracy $\textrm{ACC} = (\textrm{T}_\mathrm{{PT}} + \textrm{T}_\mathrm{{LN}})/(\textrm{T}_\mathrm{{PT}} + \textrm{F}_\mathrm{{PT}}+ \textrm{T}_\mathrm{{LN}} + \textrm{F}_\mathrm{{LN}})$.

The analysis was performed with the R language and environment for statistical computing (version 4.2.0) [96].

### Structure-wise analysis

The ability of the network to identify and classify individual lesions was assessed via structure-wise analysis as proposed in [74]. Shortly, for each lesion in the ground truth and CNN delineation, a coverage fraction by the complementary delineation was calculated. A ground truth lesion was considered as identified (true positive with respect to manual delineation, $\text {TP}_{\text {man}}$) if it was at least 50% covered by the CNN delineation and as missed by the CNN (false negative, FN) if coverage was below 50%. CNN delineated structures with coverage over 50% were considered true positive with respect to CNN delineation ($\text {TP}_{\text {cnn}}$) and the remaining CNN delineations were not corresponding to a ground truth lesion and were therefore considered false positives (FP). Based on this classification, true positive rate $\textrm{TPR}_\mathrm{{str}} = \text {TP}_{\text {man}}/(\text {TP}_{\text {man}}\ + \textrm{FN})$ and positive predictive value $\textrm{PPV}_\mathrm{{str}} = \text {TP}_{\text {cnn}}/(\text {TP}_{\text {cnn}}\ + \textrm{FP})$ were calculated.

### Survival analysis

We also investigated the impact of the differences between manual and automated delineation on a survival analysis of the patient data. The full survival analysis of these data is the objective of the above mentioned still ongoing clinical study. In the present investigation, we therefore only have exemplary considered the prognostic value of TTB for overall survival (OS). All patients satisfying the following criteria were included into this analysis: primary chemoradiotherapy, $[^{18}$F]FDG PET/CT prior to therapy, minimum follow up time of 6 months, no distant metastases, and no surgery ($N = 585$ patients from 10 institutions in main dataset; $N = 142$ patients from 2 institutions in external test dataset). The median TTB of the manual delineation in the cross-validation data was used as cutoff value for differentiating high and low risk groups in, both, manual as well as CNN delineated lesions ($\textrm{TTB}_\mathrm{{man}}$ and $\textrm{TTB}_\mathrm{{cnn}}$, respectively). The same cutoff was also used in analysis of the external data. For all delineations, an univariate Cox regression and a Kaplan-Meier analysis was performed. Results with $p<0.05$ were considered significant.

## Results

### Spatial concordance

**Fig. 1:**
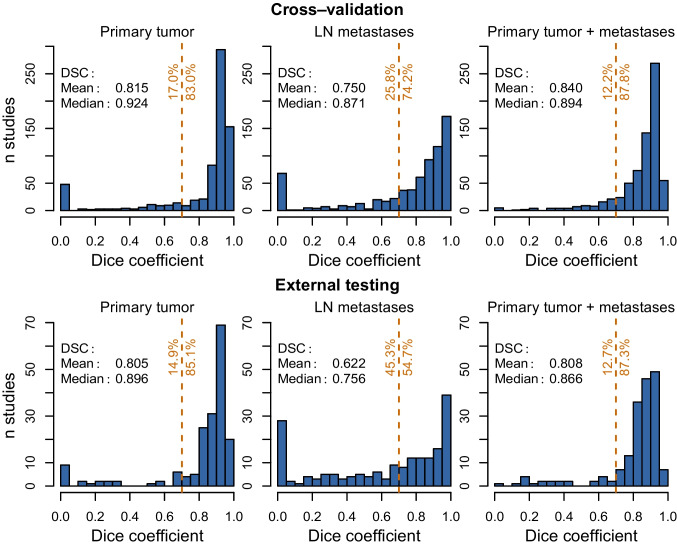
Frequency distribution of the observed Dice coefficients (CNN vs. manual delineation/labeling) for primary tumor (left), LN metastases (middle), and the union of both (right) in cross-validation (top, $N=698$ patients) and external testing data (bottom, $N=181$ patients). The dashed vertical line indicates the location of a DSC = 0.7 threshold, a value which might be considered acceptable for practical use. The numbers to the left and to the right of the line specify the percentage of cases yielding a DSC below and above that threshold, respectively

A summary of the delineation performance is given in Table 1. In the cross-validation experiment, delineation of all malignant lesions with the proposed network achieves a cohort DSC of 0.870 when not discriminating between primary tumor and lymph nodes. Treating primary tumor and lymph node metastases as distinct classes yields cohort DSCs of 0.885 and 0.805, respectively. In the external test data, cohort DSC reaches 0.850, 0.724, and 0.823 for primary tumor, LN metastases and their union, respectively. The frequency distributions of the individual respective DSCs obtained in different patients is given in Fig. 1. The delineation failed (DSC = 0) in 5 cases (0.7%) when not discriminating between primary tumor and lymph nodes. When treating primary tumor and LN metastases as distinct, delineation failed in 48 (6.9%) and 65 (9.3%) cases, respectively. In the external dataset, the delineation failed in 9 (5.0%) and 27 (14.9%) cases when treating primary tumor and LN metastases as distinct, respectively, and it never failed for the union.

**Table 1:** Delineation performance with respect to the target volume in cross-validation and external testing

Target volume	DSC				N failed ($\textrm{DSC} = 0$)
	Cohort	Mean	Median	50% CI	
Cross-validation ($N = 698$)					
Primary tumor	0.885	0.815	0.924	[0.856, 0.948]	48
LN metastases	0.805	0.750	0.871	[0.688, 0.948]	65
Primary + metastases	0.870	0.840	0.894	[0.814, 0.929]	5
External testing ($N = 181$)					
Primary tumor	0.850	0.805	0.896	[0.827, 0.926]	9
LN metastases	0.724	0.622	0.756	[0.307, 0.930]	27
Primary + metastases	0.823	0.808	0.866	[0.803, 0.909]	0

**Fig. 2:**
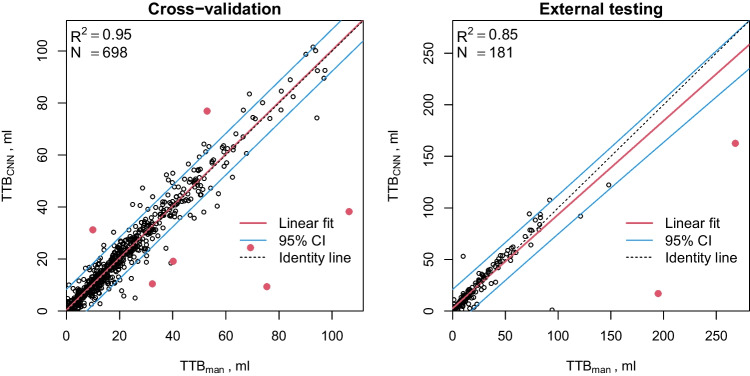
Correlation between manually and automatically derived total tumor burden (TTB: sum of primary tumor and LN metastases) in the cross-validation (left) and external testing (right) data. Note the difference in scale between the plots. Solid red points indicate outliers, defined as data points where the deviation of CNN from manual delineation exceeds the 99% percentile (i.e., the top 1%). These outliers were excluded from regression analysis. The red line represents the least squares fit of a straight line to the remaining data. The blue lines delineate the corresponding 95% prediction (tolerance) interval of expected scatter of individual data points around the regression line

Figure 2 demonstrates the degree of correlation between the manually and automatically derived TTB in, both, cross-validation and external testing experiments ($R^2 = 0.95$ and $R^2 = 0.85$, respectively, excluding the outliers). The mean absolute TTB difference was $2.62$ ml and $4.29$ ml in cross-validation and external testing data, respectively.

### Classification capabilities

In cross-validation data, the overall classification accuracy was 98.0%. 640 of the scans (91.7%) did not exhibit any classification errors. In external data, the classification accuracy was 97.9% with 147 of the scans (81.2%) free of any classification errors. The corresponding true positive rates for primary and LN metastases classification and the full contingency tables are given in Table 2.

**Table 2:** Classification performance in cross-validation and external testing. The contingency tables are normalized so that the sum over the respective table’s elements equals 1 (100%)

Dataset	Contingency table				$\textrm{TPR}_\mathrm{{PT}}$	$\textrm{TPR}_\mathrm{{LN}}$	ACC
	$\textrm{T}_\mathrm{{PT}}$	$\textrm{F}_\mathrm{{PT}}$	$\textrm{T}_\mathrm{{LN}}$	$\textrm{F}_\mathrm{{LN}}$			
Cross-validation (801384 voxels)	60.8%	1.5%	37.2%	0.5%	99.2%	96.1%	98.0%
External testing (229448 voxels)	67.0%	1.2%	30.9%	0.9%	98.7%	96.3%	97.9%

### Structure-wise analysis

In cross-validation data, in 44/252 cases non-pathological uptake was delineated and marked as primary tumor/LN metastases (false positives). 65 primaries and 229 LNs were not recognized by the network (false negatives). In external data (primary/LN), 10/53 false positive delineations were produced and 11/204 lesions were missed by the CNN. The complete statistics as well as the network’s $\textrm{PPV}_\mathrm{{str}}$ and $\textrm{TPR}_\mathrm{{str}}$ is provided in Table 3.

**Table 3:** Lesion detection performance with respect to the target volume in cross-validation and external testing

Target volume	$\text {TP}_{\text {man}}$	$\text {TP}_{\text {cnn}}$	FN	FP	$\textrm{TPR}_\mathrm{{str}}$	$\textrm{PPV}_\mathrm{{str}}$
Cross-validation ($N = 698$)						
Primary tumor	578	592	65	44	89.9%	93.1%
LN metastases	849	893	229	252	78.8%	78.0%
Primary + metastases	1427	1485	294	296	82.9%	83.4%
External testing ($N = 181$)						
Primary tumor	164	160	11	10	93.7%	94.1%
LN metastases	193	198	204	53	48.6%	78.9%
Primary + metastases	357	358	215	63	62.4%	85.0%

### Examples

**Fig. 3:**
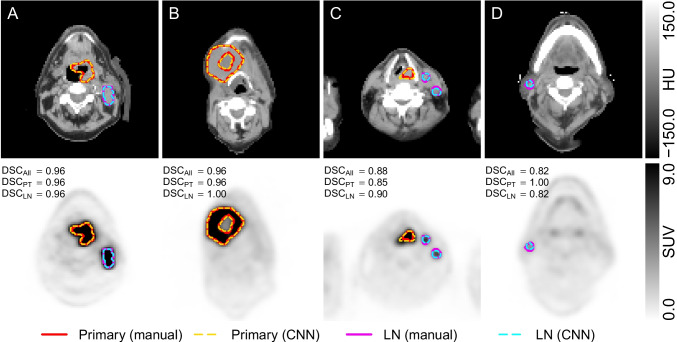
Manual and CNN-based delineations of primary tumor and lymph node metastases in 4 selected patients. Relevant transaxial PET/CT slices are shown (top: CT, bottom: PET). The dice coefficients (in the presented plane) for primary tumor ($\text {DSC}_{\text {PT}}$), LN metastases ($\text {DSC}_{\text {LN}}$), and their union ($\text {DSC}_{\text {All}}$) are indicated. Patient A: oropharyngeal cancer with LN metastasis; patient B: oropharyngeal cancer with necrotic core; patient C: hypopharyngeal cancer with 2 LN metastases; patient D: cancer of oral cavity (not visible in this slice) exhibiting a low uptake LN metastasis

Figure 3 shows exemplary delineations of four patients (A-D) from the cross-validation test subset. Patient A demonstrates that the trained CNN is able to accurately determine the contours of both primary tumor and LN metastasis and to correctly classify them as such. Patient B demonstrates the ability of the CNN to delineate relatively large lesions. Note that the human observer and the CNN consistently excluded the necrotic core of the tumor from MTV delineation. Patient C demonstrates that the CNN is able to delineate multiple structures exhibiting different relative contrast ($\text {SUV}_{\text {max}} = 22.0$ vs $\text {SUV}_{\text {max}} = 7.4$ for primary tumor and LN metastases, respectively) simultaneously. Patient D illustrates the CNN’s capability to distinguish small lesions with low uptake ($\text {SUV}_{\text {max}} = 4.0$) from comparable physiological focal uptake in other regions of the same image.

**Fig. 4:**
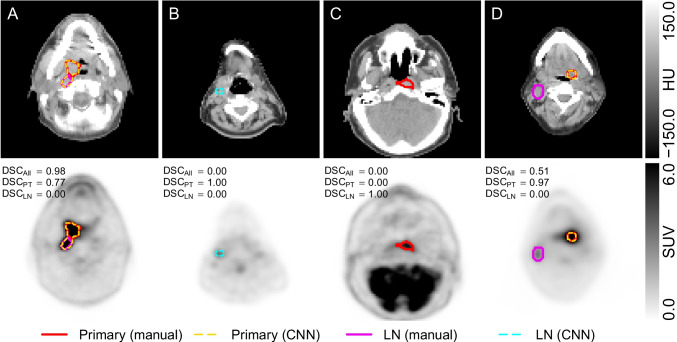
Examples of CNN delineation errors in 4 selected patients. Relevant transaxial PET/CT slices are shown (top: CT, bottom: PET). The dice coefficients (in the presented plane) for primary tumor ($\text {DSC}_{\text {PT}}$), LN metastases ($\text {DSC}_{\text {LN}}$) and their union ($\text {DSC}_{\text {All}}$) are indicated. Patient A with laryngeal cancer and multiple LN metastases (only one in plane): LN metastasis incorrectly classified as primary; patient B with oropharyngeal cancer and LN metastasis (both out of plane): CNN produced spurious LN metastasis ROI; patient C with nasopharyngeal cancer and LN metastases (out of plane): primary missed by CNN; patient D with oropharyngeal cancer and low and diffuse uptake LN metastasis: LN metastasis missed by CNN

Figure 4 shows example cases where CNN delineation failed for some lesions. In example A, the LN metastasis was misclassified as primary tumor. In example B, the CNN produced a spurious LN metastasis ROI. In examples C and D, the CNN missed the primary tumor and LN metastasis, respectively.

### Survival analysis

**Fig. 5:**
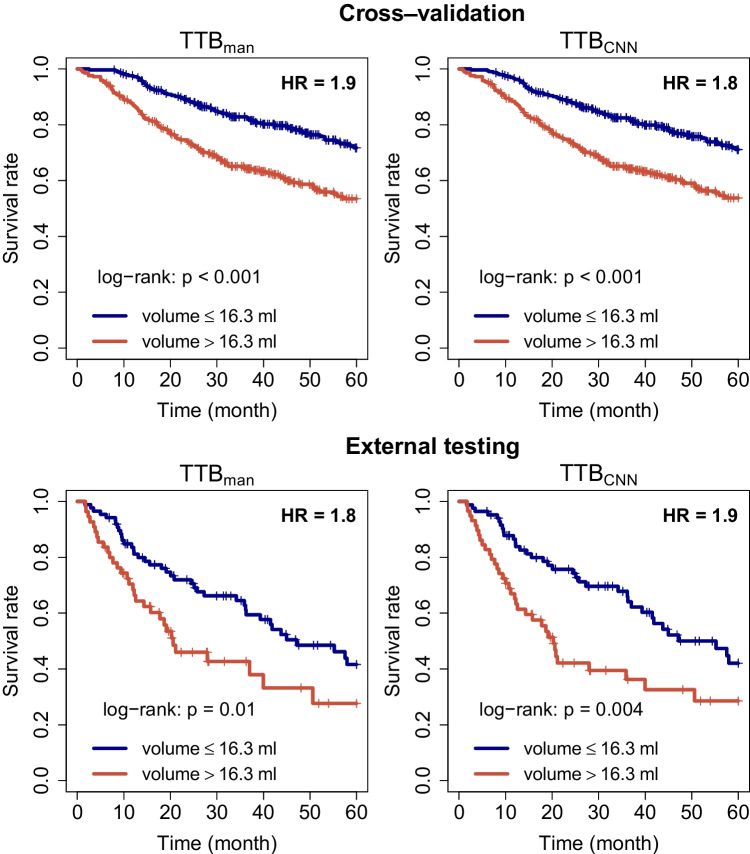
Kaplan-Meier curves with respect to OS in cross-validation (top) and external testing (bottom) data

Univariate Cox regression in the cross-validation data consistently revealed $\textrm{TTB}_\mathrm{{man}}$ as well as $\textrm{TTB}_\mathrm{{cnn}}$ as highly prognostic factors for OS with practically identical hazard ratios (HR) ($\text {HR}=1.9$; $p<0.001$ and $\text {HR}=1.8$; $p<0.001$, respectively). In 5.3% of the cases binarization led to a different classification, where classification was incorrect in 2.6% for $\textrm{TTB}_\mathrm{{man}}$ and in 2.7% for $\textrm{TTB}_\mathrm{{cnn}}$. Both, $\textrm{TTB}_\mathrm{{man}}$ and $\textrm{TTB}_\mathrm{{cnn}}$ prognostic factors also reached significance in external data and exhibited virtually the same hazard ratios as in the cross-validation dataset ($\text {HR}=1.8$; $p=0.011$ and $\text {HR}=1.9$; $p=0.004$, respectively). The fraction of cases resulting in different classification was higher in the external dataset than in the cross-validation one reaching 7.7%, where classification was incorrect in 5.6% for $\textrm{TTB}_\mathrm{{man}}$ and in 2.1% for $\textrm{TTB}_\mathrm{{cnn}}$. The corresponding Kaplan-Meier curves are shown in Fig. 5.

## Discussion

In this investigation we have demonstrated that fully automated simultaneous delineation and classification of metabolically active lesions in HNC, discriminating between primary tumor and lymph node metastases, is feasible with a suitable CNN architecture trained on combined PET/CT patient data. The achieved cohort DSC of 0.870 indicates state of the art performance of our network and is in line with the mean DSC of 0.87 reported in [87] for PET-based GTV delineation. However, differences in the used dataset and data preprocessing scheme do not allow for direct comparison between that study and our present investigation. Similarly, only indirect comparison in lesion detection performance is possible to [74] reporting mean patient-wise (TPR/PPV) of (0.86/0.33) for primary tumor + LN metastases detection as compared to cohort (TPR/PPV) of (0.83/0.83) in the present study.

As far as primary tumor delineation alone is concerned, one might validly ask whether there are practical advantages of a CNN-based delineation for tumor entities with usually high tumor to background uptake ratios such as HNC. In fact, much simpler approaches have previously been shown to work adequately in such circumstances. E.g., Ha et al. used interactively defined ellipsoidal ROIs and fixed SUV thresholds within those ROIs to successfully determine MTV and TLG in head and neck soft tissue sarcoma [86] and demonstrated the prognostic value of the such derived parameters. Regarding the data underlying the present investigation we, too, have previously resorted to a semi-automatic adaptive threshold based method developed in our group [97] for delineation in the context of the previously published clinical study [4] and obtained adequate results in about 95% of the delineations.

However, when extending the task to delineation of lymph node metastases one faces the problem that many lymph nodes exhibit only modest $[^{18}$F]FDG uptake with typical SUVs of about 3–4 (or lower). This is comparable to the level of the physiological $[^{18}$F]FDG uptake of various structures in the nasopharyngeal region, e.g., tonsils, minor salivary glands, brown adipose tissue, vocal cords or, in some cases, also muscles. In this situation, threshold based methods tend to fail and manual intervention or fully manual delineation becomes frequently necessary which is very tedious and time-consuming.

It is exactly this context of delineation at low target to background ratios where the CNN-based approach proves to be distinctly superior. This has recently also been demonstrated by Han and coworkers [98] in a different tumor entity (thymic epithelial tumor) which seems to pose a challenge comparable to the one encountered in the present study regarding the lymph node metastases.

However, the distinguishing advantage of the presently proposed approach is its ability to not only provide decent delineation for, both, high and low contrast structures but also to perform fully automated identification of primary tumor and LN metastases thus providing additional classification information for inclusion into further analysis.

The level of concordance between CNN and the human observer provided ground truth in the present study is superior to typically encountered human interobserver concordance which has, e.g., been reported in [99] as DSC = 0.69 for GTV delineation in PET/CT. Although interobserver concordance might be expected to be somewhat higher for MTV delineation [100], experience tells that it generally will not exceed the degree of concordance between CNN and human observer reported in the present study. This can be rephrased as stating that our trained network overall is capable to perform mostly comparably to an experienced human reader (specifically, the reader(s) having provided the ground truth delineation used in training the network). While it is not able to replace said human observer (due to the remaining sporadic incidences of failure), it is well suited to be utilized as an efficient delineation assistance tool in clinical and research contexts. As has also been reported elsewhere, utilizing such tools will considerably reduce (without manual intervention: eliminate) interobserver variability while also providing obvious speed benefits compared to fully manual or semi-automatic delineation [101]. We believe that the presently proposed CNN especially has potential to facilitate large-scale clinical study evaluations and in the next step could allow to utilize translation of findings from such studies to the clinical routine without imposing intolerable time demands on the clinician.

For example, in the present study the native CNN-based TTB determination yielded outcome predictions for HNC patients (regarding overall survival) of a quality fully competitive to and concordant with prediction based on manually derived TTBs (Fig. 5). This observation can be traced back to the very decent correlation between manual and CNN-based TTB volumes ($R^2=0.95$) and the low number of definite outliers as demonstrated in Fig. 2. Further added value is provided by the network’s classification capabilities allowing to derive metabolic volumes separately for primary tumor and lymph node metastases which provides the prerequisite to further tailor the decision support process for specific cancer types [102, 103].

A fundamental concern regarding adoption of deep learning approaches in diagnostic imaging and data evaluation is a possible inability of the trained network to generalize from training data to new, so far unseen data [104]. It is theoretically conceivable that the trained CNN actually has incorporated (“learned”) very specific inherent characteristics of the training data and requires their presence in any new input in order to perform successful delineation. A similar problem appears in the context of radiomics where it is addressed via data harmonization procedures [105, 106]. Consequently, it has been suggested to use data harmonization to tackle the generalization problem in CNN-based delineation as well [84]. However, there is currently not enough evidence supporting the usefulness of this strategy. In the present study we have approached the problem from the opposite direction: rather than aiming at harmonization of the training data as well as any “new” data to which the trained network should be applied, we intentionally included “heterogeneous” training data from as many varied sources and institutions as we found doable. Altogether, we were able to collect 698 scans from eight independent data sources. Our working hypothesis was that exposing the network to images with different image characteristics would force the CNN to learn common properties of the images and promote generalization. This hypothesis was then tested in an additional independent external dataset. Even though the overall DSC decreased from 0.870 in the cross-validation results to 0.823 in these external data, it remained high enough to prove good generalizability of the developed network. Survival analysis confirms this conclusion revealing that automatically derived TTB remains prognostic of overall survival in external data, too, with virtually identical hazard ratio to those observed in both manual delineation and cross-validation data. The main driver for reduction in overall DSC was reduced performance of LN metastases delineation ($\text {DSC}_\text {LN} = 0.724$ vs $\text {DSC}_\text {LN} = 0.805$ in external and cross-validation data, respectively). This behavior can be potentially explained by the differences in the distribution of volumes of LN metastases across the datasets (median volume = 2.74 ml vs 0.70 ml, in main and external datasets, respectively) as smaller lesions are generally more difficult to detect and unambiguously delineate.

The representative examples shown in Fig. 3 demonstrate that the trained CNN is principally able to provide fully satisfactory MTV delineation across a wide range of image characteristics regarding tumor and LN metastases location and target/background contrasts. As Fig. 3 B demonstrates, the network also is able to correctly delineate diverse tumor shapes and to exclude necrotic tumor areas. Figure 3 D demonstrates the arguably most important capability of a CNN-based delineation approach, namely the ability to differentiate elevated “physiological” uptake from malignant lesions.

However, despite satisfactory performance in the majority of cases (obviating any need for manual correction), delineation and classification errors of different severity including occasional manifest failure were also observed. Figure 4 demonstrates some of the most common patterns.

Figure 4 A shows an instance of misclassification of LN metastasis and primary tumor which is reflected in correspondingly reduced DSCs. Such partial misclassification does not affect the TTB parameter at all and can be rather easily corrected manually if deemed necessary. The latter is also true for a related type of error, namely misclassification of physiological focal uptake (e.g., inflammation) as malignant lesion as shown in Fig. 4 B. Actually, this concerns a considerable fraction (22.0%) of the generated ROIs for LN metastases. This can intuitively be understood as a consequence of the fact that inflamed and metastatic LN cannot be discriminated unambiguously based on the image data alone. Discrimination between them by the clinician usually is based on additional clinical information that is not accessible to the CNN. This type of error, too, is relatively easy to correct manually by deleting the spurious ROIs but it occurs in a notable fraction of all cases (25.6%) and will therefore contribute noticeably to the remaining time demands required for user intervention when performing CNN-assisted delineation.

Figure 4 C and D demonstrate instances of failure to identify the primary tumor (C) or LN metastases (D). For such cases, manual intervention/delineation would obviously be required. Such failures occurred in 9.3% (22.2%) of the patients for the primary tumor (LN metastases). However, the mean volume of the missed lesions was 8.16 and 2.78 ml for primary tumor and LN metastases, respectively, which is almost a factor of two lower than the respective mean volumes of 13.21 and 5.45 ml of all lesions, suggesting that mainly small lesions were not detected. In the affected patients, the missed lesions contributed on average 33.6% (median: 16.9%) of the ground truth TTB, indicating that their impact on the derived TTB values was limited in most of the cases, however large errors also occurred.

Due to the black box nature of neural networks, it is inherently difficult to identify specific image characteristics that tend to cause misdelineation/classification. What we have noticed is that sizable errors predominantly occurred for tumors of unusual composition (e.g., Fig. 4 A, where a seemingly singular lesion is in fact the primary tumor and the LN metastasis in direct vicinity of each other) or localization (Fig. 4 C, nasopharyngeal cancer contributed only 6% of the cases in the main dataset). Furthermore, differentiation between malignant and benign $[^{18}$F]FDG-positive LNs is complicated even for experienced human observers, particularly in the cases of low (Fig. 4 B) or diffuse (Fig. 4 D) uptake and small lesion size.

Consequently, in such circumstances the ground truth manual delineation and classification itself is not completely unambiguous which inherently limits the obtainable degree of concordance between different observers (either human or neural network). This is our tentative explanation for the observation that overall concordance between network and our ground truth delineation was better for the primary tumors than for lymph nodes. In this context, it has also to be noted, that ground truth definition was based on a single manual delineation per lesion which constitutes an obvious limitation of the present investigation.

In fact, it is quite likely that the increase of training data afforded by multiple independent delineations for each patient would further improve the performance of the resulting network. However, recruiting further experienced observers for the very time-intensive task of performing hundreds of delineations was not feasible within the limits of the present investigation.

Another potential limitation is the omission of data augmentation frequently employed to prevent overfitting which turned out not to be doable due to limited capabilities of the utilized deep learning framework MXNet when dealing with 3D image volumes (affine and warp transforms not available). We compensated for this deficiency by heavy sampling of all 3D data sets with 75% overlap between patches which effectively functions as image shift augmentation. The comparatively large number of tomographic data sets available for network training within this study should further reduce the benefits of additional data augmentation.

## Conclusion

To the best of our knowledge, this study presents the first CNN for simultaneous MTV delineation and lesion classification for $[^{18}$F]FDG PET/CT in HNC patients. Our network allows fast delineation and classification of primary tumor and lymph node metastases in HNC while rarely requiring more than minimal manual corrections. It thus is a capable tool able to massively accelerate and facilitate study data evaluation in large patient groups which also does have clear potential for supervised clinical application.

## Supplementary Information

Below is the link to the electronic supplementary material.

Supplementary file1 (PDF 211 KB)

## Data Availability

Sources of data from public databases are listed in the “Methods’’ section. All other data are available from the institutes contributing the respective data to the present study upon reasonable request. The developed mxnet CNN models are available from the authors upon reasonable request.
