# Supplementary material for: A convolutional neural network with self-attention for fully automated metabolic tumor volume delineation of head and neck cancer in [18F]FDG PET/CT
Source: Eur J Nucl Med Mol Imaging. 2023 Apr 20;50(9):2751–66. doi: 10.1007/s00259-023-06197-1 (PMC10317885; doi:10.1007/s00259-023-06197-1)
Supplement: Supplementary file 1 — Supplementary file1 (PDF 211 KB) [file 259_2023_6197_MOESM1_ESM.pdf]

European Journal of Nuclear Medicine and Molecular Imaging

Supplementary material:

A convolutional neural network with self-attention for fully automated metabolic tumor volume delineation of head and neck cancer in PET/CT

Pavel Nikulin [1,*,†], Sebastian Zschaeck [2,*], Jens Maus [1], Paulina Cegła [3], Elia Lombardo [4], Christian Furth [5], Joanna Kaźmierska [6,7], Julian Rogasch [5], Adrien Holzgreve [8], Nathalie L. Albert [8], Konstantinos Ferentinos [9], Iosif Strouthos [8], Marina Hajiyianni [2], Sebastian N. Marschner [4], Claus Belka [4,10], Guillaume Landry [4], Witold Cholewinski [3,6], Jörg Kotzerke [11], Frank Hofheinz [1], Jörg van den Hoff [1,11]

[1] *Helmholtz-Zentrum Dresden-Rossendorf, PET Center, Institute of Radiopharmaceutical Cancer Research*
[2] *Charité – Universitätsmedizin Berlin, corporate member of Freie Universität Berlin, Humboldt-Universität zu Berlin, and Berlin Institute of Health, Department of Radiation Oncology, Berlin, Germany, Berlin Institute of Health, Berlin, Germany*
[3] *Department of Nuclear Medicine, Greater Poland Cancer Centre, Poznan, Poland*
[4] *Department of Radiation Oncology, University Hospital, Ludwig-Maximilians-University (LMU) Munich, Munich, Germany*
[5] *Department of Nuclear Medicine, Charité – Universitätsmedizin Berlin, corporate member of Freie Universität Berlin, Humboldt-Universität zu Berlin, and Berlin Institute of Health, Berlin, Germany*
[6] *Electroradiology Department, University of Medical Sciences, Poznan, Poland*
[7] *Radiotherapy Department II, Greater Poland Cancer Centre, Poznan, Poland*
[8] *Department of Nuclear Medicine, University Hospital, Ludwig-Maximilians-University (LMU) Munich, Germany*
[9] *Department of Radiation Oncology, German Oncology Center, European University Cyprus, Limassol, Cyprus*
[10] *German Cancer Consortium (DKTK), Partner Site Munich, Munich, Germany*
[11] *Department of Nuclear Medicine, University Hospital Carl Gustav Carus, Technische Universität Dresden, Dresden, Germany*

Neural network architecture

Automated lesion delineation was performed with a modified residual 3D U-Net CNN, see fig. 1. The network consists of encoder and decoder paths each of which represents an alternating sequence of residual blocks and, respectively, downsampling ($2 \times 2 \times 2$ max-pooling) and upsampling ($3 \times 3 \times 3$ deconvolution) steps. The residual block combines $3 \times 3 \times 3$ convolution, batch normalization, leaky ReLU and dropout (rate $= 0.1$) layers and applies them to the input twice in a row while also providing a bypass skip-connection to improve the gradient flow and accelerate training. Since the number of input and output features can differ, $1 \times 1 \times 1$ convolution is used in the skip-connection for equalization.

Features identified by the encoder are transferred to the decoder via skip-connections and concatenated with the corresponding decoder features at all image scales except the lowest one. At the bottom of the U-Net the encoder and decoder are connected through a Multi-Head Self-Attention (MHSA) block to improve awareness of the global context in classification of FDG-avid regions. MHSA was first introduced in [1] for natural language processing and was further adapted for image processing. Our implementation of MHSA mostly follows [2] but includes a few modifications. We refer to [2] for further details on, and motivation behind, MHSA while here we only provide a short overview of the method.

In our implementation, the MHSA block comprises two Self-Attention (SA) heads which share the same input. Each SA head establishes a rule according to which the information is transferred across the whole image. The rule is defined via projection operators (implemented as $1 \times 1 \times 1$ convolution) of the input

* P. Nikulin and S. Zschaeck contributed equally to this article.
† Bautzner Landstrasse 400, 01328 Dresden, Germany. E-mail: p.nikulin@hzdr.de

tensor into queries, keys, and values matrices: $\mathbf{Q} \in \mathbb{R}^{n\times k}$, $\mathbf{K} \in \mathbb{R}^{n\times k}$, and $\mathbf{V} \in \mathbb{R}^{n\times k}$, respectively. Here, $n = 16 \times 16 \times 4$ is the total number of voxels in the input tensor and $k = 64$ is the number of dimensions of the projection space. This way, each voxel i is represented by vectors $\mathbf{q}_i$, $\mathbf{k}_i$, and $\mathbf{v}_i$ of length k which are stored as lines of the respective matrices. Values vector $\mathbf{v}_j$ holds the relevant information about voxel j which is going to be transferred while vectors $\mathbf{q}_i$ and $\mathbf{k}_j$ determine the information transfer rate from voxel j to voxel i based on their similarity. In our implementation, we define similarity between $\mathbf{q}_i$ and $\mathbf{k}_j$ as a cosine of the angle between them calculated as a dot-product of the normalized vectors. The similarity coefficients of voxel i to every other voxel j are softmaxed and written in the form of an attention matrix $\mathbf{A} \in \mathbb{R}^{n\times n}$. Each matrix element $\mathbf{A}_{ij}$ denotes the relative importance of the voxel j for classification of voxel i. The output of the SA head for each voxel i is the sum of values $\mathbf{v}_j$ weighted with $\mathbf{A}_{ij}$:

$$\mathrm{SA}(\mathbf{Q}, \mathbf{K}, \mathbf{V}) = \mathrm{softmax}(\widetilde{\mathbf{Q}}\,\widetilde{\mathbf{K}}^T)\,\mathbf{V} = \mathbf{AV},$$

where the tilde denotes row-wise L^2-normalization of the matrix and the softmax is applied row-wise. The outputs of all SA heads are concatenated and combined together via another $1 \times 1 \times 1$ convolution (without bias).

Importantly, the SA mechanism has no means to account for the relative position of the voxels in the image. To give the SA heads access to the spatial information the input tensor is concatenated with a positional encoding tensor [1]. Moreover, we added a residual connection around the MHSA block to improve the gradient flow.

The described architecture was implemented using the `Apache MXNet` (version 1.9.0) package for the `R language and environment for statistical computing` (version 4.2.0) [3].

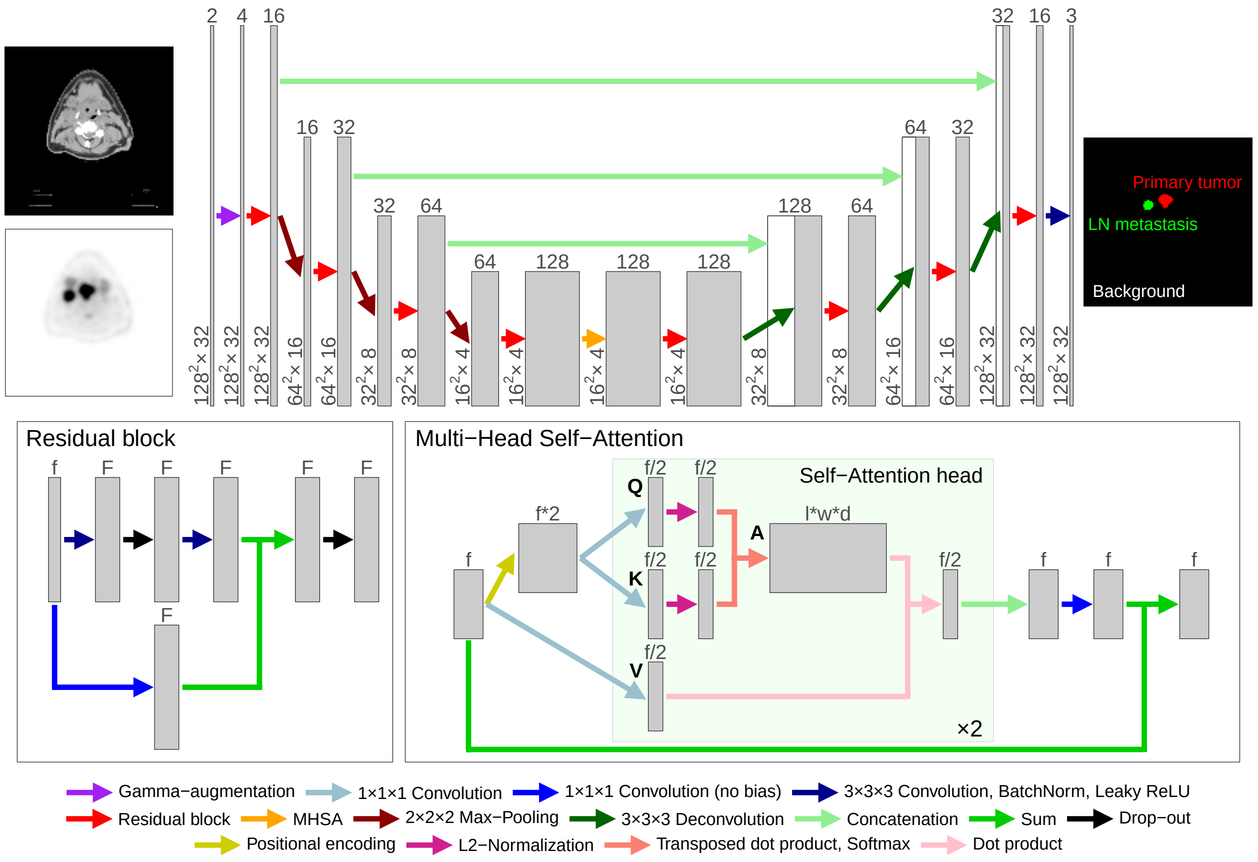

Figure 1: Architecture of the utilized CNN. Numbers above and beside each block designate the number of feature channels and matrix size at the given state, respectively. The images on the left are exemplary CT (top) and PET (bottom) images (input) and the image on the right is the corresponding output image (probability maps of the background, primary tumor, and lymph-node metastases shown in black, red, and green respectively)

Loss function and evaluation metrics

The loss function used for the network training is the sum of soft Dice and Cross-Entropy (CE) losses:

$$\mathcal{L}(p, y) = \frac{1}{2}\mathcal{L}_{Dice}(p, y) + \frac{1}{2}\mathcal{L}_{CE}(p, y), \tag{1}$$

$$\mathcal{L}_{Dice}(p, y) = -\frac{1}{C}\sum_{j=0}^{C-1} \frac{\sum_{i=0}^{N-1} 2\, p_{ij}\, y_{ij}}{\sum_{i=0}^{N-1} p_{ij} + \sum_{i=0}^{N-1} y_{ij} + \varepsilon}, \tag{2}$$

$$\mathcal{L}_{CE}(p, y) = \frac{1}{CN}\sum_{j=0}^{C-1}\sum_{i=0}^{N-1} \Big[p_{ij}\,(y_{ij} - 1)\log(1 - p_{ij}) - (1 - p_{ij})\, y_{ij}\log(p_{ij})\Big], \tag{3}$$

where y_{ij} and p_{ij} are, respectively, ground truth (binary) and predicted probabilities of voxel i belonging to class j. N is the number of voxels in the training batch and $C = 3$ is the number of classes. Here, the smoothing constant $\varepsilon = 1$ is added to the denominator in (2) for numerical stability.

The evaluation metric used for monitoring the training process in the validation data was the multiclass soft Dice function. The formula for the soft Dice metric function is similar to (2) with a difference in the expression sign and the fact that the loss function is calculated separately for each (relatively small) image batch while the evaluation metric is computed for the whole validation dataset at once. The variance of the evaluation metric was reduced in runtime using exponential smoothing (smoothing factor $\alpha = 0.6$).

Data acquisition and image reconstruction

Table 1: Data acquisition and image reconstruction parameters of the PET data. For the data from the databases this information was taken from the DICOM image data. Missing information is indicated by 'NA'.

	Main dataset	External testing
**Scanner**		
Philips, Gemini-TF	197	0
General Electric, Discovery ST(E)	168	0
Philips, Guardian Body (C)	133	0
Siemens, NA	131	0
Siemens, Biograph 16	24	0
General Electric, Discovery 690	0	119
Siemens, Biograph mCT 20	0	47
General Electric, Discovery IQ	0	15
NA	45	0
Scan duration (s)	(90 – 420)	(150 - 180)
**Reconstruction (type, iterations/subsets)**		
BLOB-OS-TF, 3i/33s	197	0
OSEM, NA	168	0
LOR-RAMLA, NA	133	0
OSEM, 2i/8s	131	0
OSEM, 4i/8s	24	0
VUE Point Fx, 2i36s	0	134
TrueX, 3i21s	0	47
NA	45	0
**Time of flight**		
Yes	197	166
No	325	15
NA	176	0
**Point spread function modeling**		
Yes	0	181
No	522	0
NA	176	0
**Image dimensions**		
Matrix size (in-plane, voxels)	(128 – 256)	(168 – 256)
Voxel size (in-plane, mm)	(2.7 – 4.1)	(2.7 – 4.1)
Voxel size (off-plane, mm)	(3.0 – 5.0)	(2.4 – 5.0)

All PET data were reconstructed including necessary attenuation, randoms, and scatter corrections. The corresponding CTs served also as input for training and testing. The matrix size of all CTs was 512x512. The in plane voxel size was (0.97 – 1.37)mm and the off plane voxel size was (2 – 5)mm. No postprocessing filter was applied to the data reconstructed with the BLOB-OS-TF method. All other data for which this information was available were postprocessed with a Gaussian filter with (2 – 5)mm FWHM. In 176 cases this information was not available.

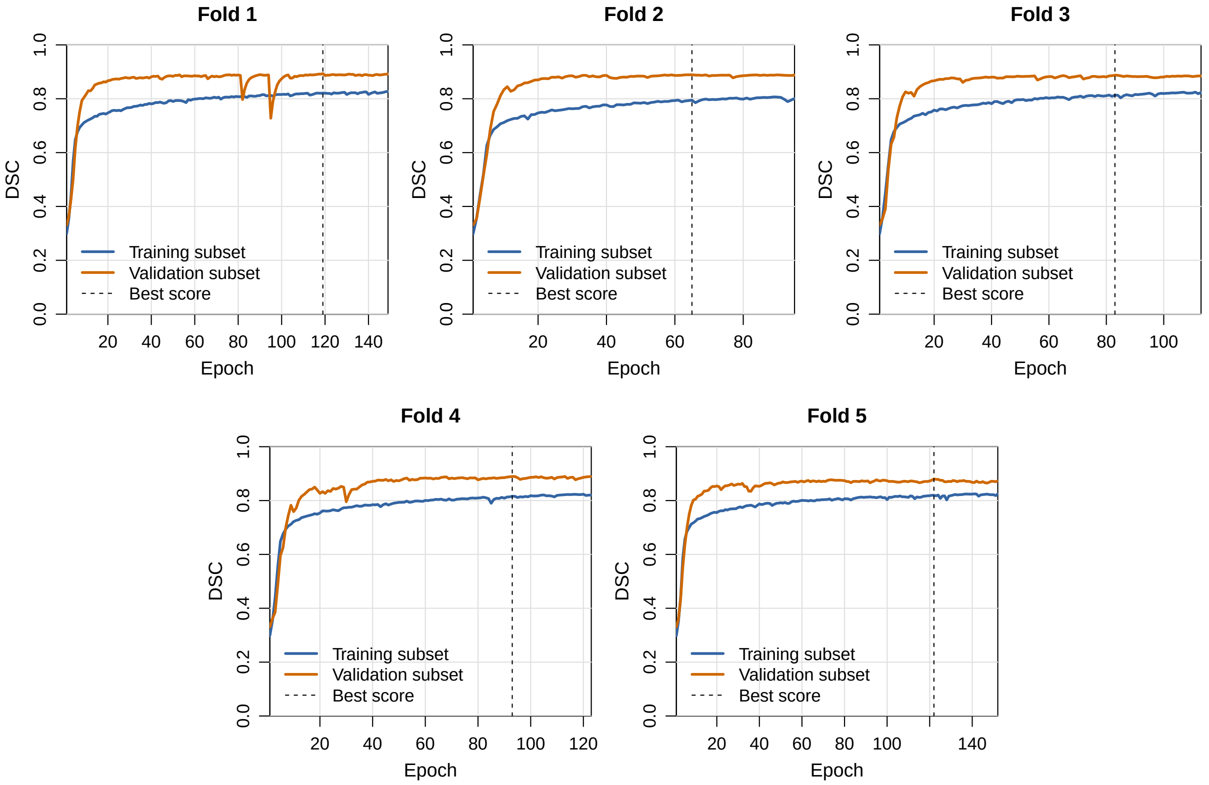

Figure 2: Logs of the training process as represented by evaluation metric (multiclass soft Dice) for training and validation subsets. Each plot corresponds to the respective split of the main dataset into training+validation and test subsets (fold). The dashed line marks the epoch for which the maximum of the evaluation metric was achieved. Note that training DSC stays generally lower than validation DSC due to dropout and data augmentations applied during the training.

References

[1] Vaswani A, Shazeer N, Parmar N, Uszkoreit J, Jones L, Gomez AN, et al. Attention is All you Need. In: Advances in Neural Information Processing Systems, Guyon I, Luxburg UV, Bengio S, Wallach H, Fergus R, Vishwanathan S, et al., editors, vol. 30. Curran Associates, Inc., 5999–6009. https://proceedings.neurips.cc/paper/2017/file/3f5ee243547dee91fbd053c1c4a845aa-Paper.pdf.

[2] Petit O, Thome N, Rambour C, Themyr L, Collins T, Soler L. U-Net Transformer: Self and Cross Attention for Medical Image Segmentation. In: Machine Learning in Medical Imaging. Springer International Publishing. 2021;267–276.

[3] R Core Team. R: A Language and Environment for Statistical Computing. R Foundation for Statistical Computing, Vienna, Austria. 2022.
